# Engineering Long‐Releasing Hollow‐like or Condensed Progesterone Hormone Microcrystals with Controlled Polymorphism

**DOI:** 10.1002/smsc.202400045

**Published:** 2024-05-26

**Authors:** Merna Shaheen‐Mualim, Edwar Odeh, Neta Kutner, Muhammad Hijazi, Shady Farah

**Affiliations:** ^1^ The Laboratory for Advanced Functional/Medicinal Polymers & Smart Drug Delivery, Technologies, The Wolfson Faculty of Chemical Engineering Technion‐Israel Institute of Technology Haifa 3200003 Israel; ^2^ The Russell Berrie Nanotechnology Institute Technion‐Israel Institute of Technology Haifa 3200003 Israel

**Keywords:** controlled release, crystals engineering, hollow‐like crystals, hormones, localized delivery, progesterone

## Abstract

Progesterone is an endogenous steroid hormone involved in the menstrual cycle, pregnancy, and embryogenesis of humans and other species. Progesterone crystallization techniques have previously reported. Among these techniques, solvent crystallization and different solvent:anti‐solvent systems are considered. Herein, the selective development of either hollow‐like or condensed progesterone microcrystals in elevated yield with controlled polymorphism, habit, and release is described for the first time. For the hollow microcrystals, isopropyl alcohol (IPA) and double‐deionized water (DDW) system is developed as solvent:anti‐solvent, while acetonitrile (AcN) and DDW system are developed for the condensed microcrystals. The microcrystals obtained from both developed crystallization systems are thoroughly investigated with varied microscopic techniques, including brightfield and scanning electron microscopy (SEM), thermal analysis by differential scanning calorimetry (DSC), and crystallography by powder X‐ray diffraction (PXRD) and single XRD, and have been compared. Results show that the crystals of the IPA:DDW crystallization system are hollow and exhibit several habits, whereas the microcrystals of the AcN:DDW crystallization system are more condensed. However, both systems are found to have a wide crystal size distribution of one stable polymorph and are thus highly useful for tunable release. More importantly, these microcrystals exhibit elongated and slow release for 14 days under an expedited release conditions model, indicating suitability for long‐term and potential localized release applications.

## Graphical abstract

Progesterone hormone has several clinical applications, including as a treatment in cases of recurrent miscarriage, late pregnancy, in vitro fertilization technologies, hormone replacement therapy, and birth control as a contraceptive agent. Herein, for the first time, the engineering and formulating of two carrier‐free crystalline structure forms of progesterone, hollow‐like and condensed microcrystals with single polymorph. Both forms show tunable release profiles and exhibit potential for localized and long‐term delivery applications.

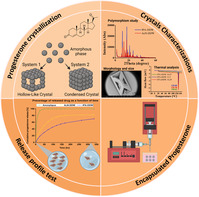

## 1 Introduction

**Figure 1:**
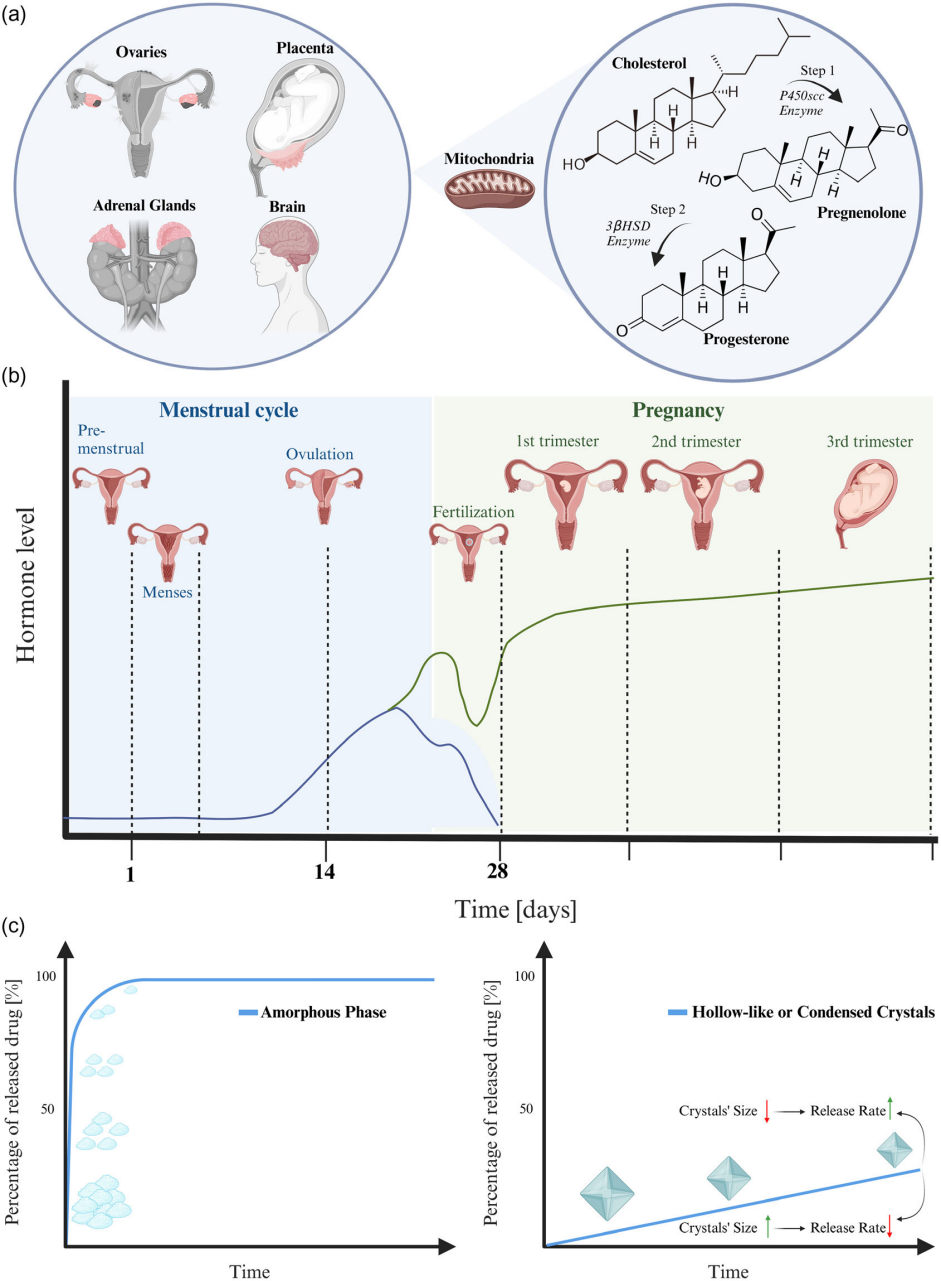
a) Progesterone production approaches by the ovaries, adrenal glands, placenta, and brain, and its synthesis from cholesterol. The biosynthesis process is catalyzed by two enzymatic steps: first by conversion of the cholesterol to pregnenolone in the mitochondria via the cholesterol side‐chain cleavage enzyme P450scc, and then by conversion from pregnenolone to progesterone via the enzyme 3β‐hydroxysteroid dehydrogenase (3β‐HSD).^[^ ^1^ ^]^ b) The hormonal change during a woman's life cycle, described by the plasma concentration of the ovarian hormone, progesterone, during the menstrual cycle (blue profile) and pregnancy (green profile) over a woman's lifetime as a function of a woman's reproductive stage. c) The rapid releasing of progesterone from the amorphous phase (*left*) versus the proposed strategy for the hollow‐like or condensed crystals (right) and how to manipulate the releasing profile to achieve the desired long‐term release. Created with BioRender.com.

Progesterone is a 21‐carbon steroid that plays a central role in female reproductive events. It also plays a vital role in several tissues that do not belong to the reproductive system, such as the central nervous and cardiovascular systems, bones, and mammary glands, in preparation for breastfeeding.^[^ ^1^ ^]^ Progesterone is predominantly produced by the ovaries, and it can also be synthesized in the placenta, adrenal glands, and the brain. Its biosynthesis is relatively simple and consists of two enzymatic steps, as shown in **Figure** **1**a.^[^ ^1^ ^]^ Progesterone is a crucial element of the menstrual cycle, both during the peri‐ and postovulatory phases, and an essential hormone associated with pregnancy establishment and maintenance. It prepares the uterus for implantation of the fertilized ovum.^[^ ^2^ ^]^ Its levels fluctuate throughout the menstrual cycle and pregnancy, as shown in Figure 1b.^[^ ^3^ ^]^

Progesterone and other progestational agents have several clinical applications. It is used as a treatment in cases of recurrent miscarriage, maintenance of uterine quiescence in late pregnancy, in vitro fertilization technologies, hormone replacement therapy, and birth control as a contraceptive agent.^[^ ^2^ ^]^ Progesterone is a poorly water‐soluble drug. Therefore, several administration routes were developed, e.g., orally, transvaginal, and subcutaneously. Vast research has been conducted to evaluate the physiological and side effects of exogenous progesterone administration, showing that local administration of progesterone has the best bioavailability and fewer side effects among the different routes.^[^ ^2^ ^]^

To enable the local, long‐term controlled release of progesterone for better medical administration, we sought to develop controlled carrier‐free microcrystals. Previously, we have reported the crystallization of various drugs from miscible solvent:anti‐solvent mixtures for the development of carrier‐free drug delivery systems based on either single crystals^[^ ^4^, ^5^ ^]^ or based on crystalline coatings.^[^ ^6^, ^7^ ^]^ As a drug release system, these crystalline drug formulations exhibited several advantages: low body immune response since they are carrier‐free,^[^ ^4^, ^5^ ^]^ high drug content where the crystals are composed 100% from the drug itself,^[^ ^4^, ^6^, ^7^ ^]^ and slow release by crystals’ surface dissolution,^[^ ^4^, ^6^, ^7^ ^]^ which can be tuned by controlling the crystals’ habit, size, and polymorphism.^[^ ^5^, ^6^, ^7^ ^]^ Progesterone crystals were thoroughly investigated and noted in the literature.^[^ ^8^, ^9^, ^10^, ^11^, ^12^, ^13^, ^14^, ^15^, ^16^, ^17^, ^18^, ^19^, ^20^, ^21^ ^]^ These studies report the existence of five forms, where two of them, forms 1 and 2, could be characterized, while the additional three were detected under hot‐stage microscopy at equilibrium melt and could not be isolated for further characterization.^[^ ^8^, ^10^, ^12^, ^16^ ^]^ Progesterone form 1 is stable owing to the hydrogen bond complexes within the molecules in the lattice, and it melts at 129–131 °C, and form 2 is unstable due to the existence of only one hydrogen bond between the molecules within the lattice and melts at 121–123 °C.^[^ ^8^, ^9^, ^20^ ^]^ Multiple studies were conducted to produce form 2 of progesterone since it was found to be more water soluble with a higher dissolution rate and hence more orally bioavailable.^[^ ^8^, ^9^, ^20^ ^]^ The formation of form 2 was enhanced using different crystallization techniques, including shear‐assisted sonocrystallization,^[^ ^8^ ^]^ melt sonocrystallization,^[^ ^9^ ^]^ cocrystallization,^[^ ^11^ ^]^ solvent rapid cooling or solvent evaporation from diluted solutions,^[^ ^16^, ^17^, ^18^ ^]^ melt crystallization,^[^ ^16^, ^17^ ^]^ and crystallization upon polymeric matrixes.^[^ ^20^ ^]^ Araya‐Sibaja et al. studied progesterone crystallization upon different polymeric matrixes and the relationship between polymorphism and crystal morphology.^[^ ^20^ ^]^ Their scanning electron microscopy (SEM) images show different morphologies of the same polymorph and even the same morphology for both polymorphs.^[^ ^17^, ^20^ ^]^ In different from reported literature, including our own reported carrier‐free studies, here, we present for the first time the engineering and formulating of two carrier‐free crystalline habits of progesterone, hollow‐like and condensed microcrystals, and thoroughly study their features, polymorphism, and suitability for long‐term release, as the proposed strategy shown in Figure 1c.

One of the widely used crystallization techniques in the pharmaceutical industry is solvent:anti‐solvent crystallization.^[^ ^5^ ^]^ Examples of a solvent:anti‐solvent crystallization systems that have been reported for the production of fine‐quality crystals of progesterone are isopropanol (IPA) and double‐deionized water (DDW),^[^ ^13^ ^]^ acetone:DDW,^[^ ^14^ ^]^ ethanol:DDW, and ethanol‐n‐hexane.^[^ ^15^ ^]^ Ragab et al. investigated progesterone microcrystals either from IPA:DDW with very low drug concentrations (0.5–1.0 mg mL^−1^) and anti‐solvent (DDW) addition in varied constant rates (10–100 mL min^−1^) or by the combination of both anti‐solvent and cooling crystallization techniques for short periods of time up to 20 min. In this study, they aimed for a narrow distribution of low micron size range crystals (≈5 μm) for pulmonary drug delivery. They reported that the small microcrystals size enables their potential use for respiratory drug delivery and increases their solubility due to the high ratio of surface to volume. However, the study did not include the crystal dissolution rate under physiological conditions, which is expected to be rapid and inappropriate for the medical case addressed in the current study.

In this work, we suggest controlling the progesterone release profile to fit the desired progesterone levels by controlling the crystal's habit, size, and size distribution of the stable polymorph prepared by solvent:anti‐solvent crystallization methodology. Therefore, indifferent from the previous works, here, we present a new modified initial progesterone concentration range of the previously reported IPA:DDW crystallization system and varied solvent:anti‐solvent ratios at constant temperature to obtain larger crystals with wider crystals’ size distribution, in addition to unprecedented solvent:anti‐solvent crystallization system utilizing an acetonitrile (AcN):DDW system to report for the first time hollow‐like and condensed progesterone microcrystals, respectively. These systems revealed a higher yield of progesterone microcrystals with the same polymorph and identified with controlled long‐term release.

## 2 Results and Discussion

**Figure 2:**
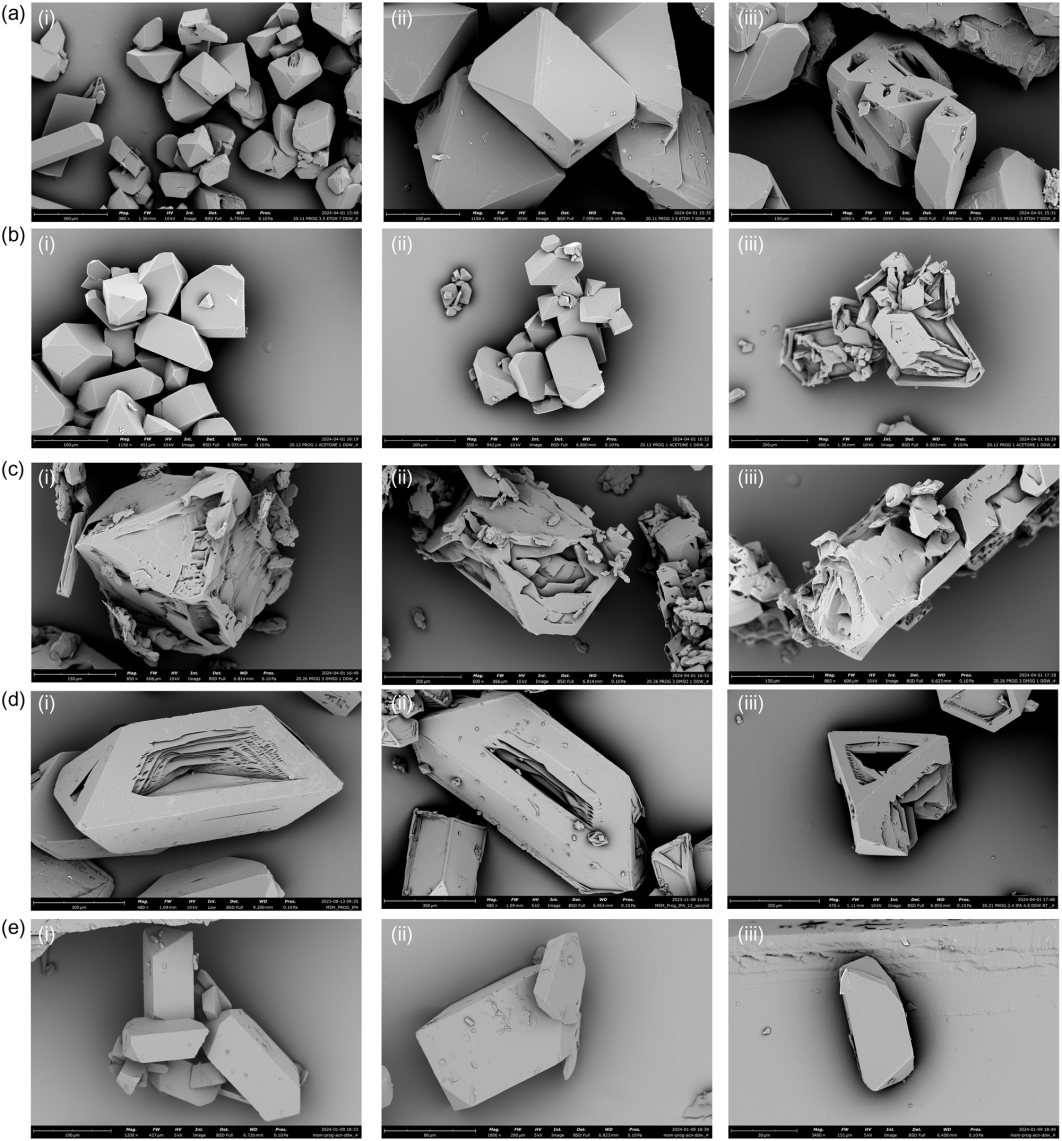
SEM images for progesterone crystals obtained from different solvent:anti‐solvent systems, where a,i–iii) represent crystals obtained from EtOH:DDW (1:2) with an initial progesterone concentration of 5.71 [mg mL^−1^], b,i–iii) represent crystals obtained from acetone:DDW (1:1) with an initial progesterone concentration of 20.00 [mg mL^−1^], c,i–iii) represent crystals obtained from DMSO:DDW (3:1) with an initial progesterone concentration of 6.66 [mg mL^−1^], d,i–iii) represent crystals obtained from IPA:DDW (1:2) with an initial progesterone concentration of 12.50 [mg mL^−1^], and e,i–iii) represent crystals obtained from AcN:DDW (1:2) with an initial progesterone concentration of 16.67 [mg mL^−1^]. Each (i–iii) denotes different crystals obtained from the specified system.

To develop the desired long‐releasing crystals with controlled habit features, we have extensively studied several miscible solvent mixtures and ratios to develop solvent‐free progesterone microcrystals. We initiated our experiments by first quantifying the solubility limit of progesterone in various organic solvents, Table S1, Supporting Information. Then, as specified in the methods section we started the crystallization methodology  by first dissolving progesterone in the solvent, followed by the addition of the anti‐solvent and crystallizing at a constant temperature. We studied various solvent:anti‐solvent systems to prepare progesterone crystals. Among the investigated systems, the solvents were ethanol (EtOH), acetone, dimethyl sulfoxide (DMSO), IPA, and AcN, each in combination with DDW as an anti‐solvent, **Figure** **2** and Table S2, Supporting Information. For EtOH:DDW system with an initial progesterone concentration of 5.71 [mg mL^−1^], progesterone was dissolved in EtOH and then DDW was added as an anti‐solvent. This system yielded crystals in a low yield, with very few hollow structures. A similar outcome was observed with the acetone:DDW system with an initial progesterone concentration of 20.00 [mg mL^−1^], where the proportion of hollow crystals remained relatively low. In the case of the DMSO:DDW system with an initial progesterone concentration of 6.66 [mg mL^−1^], the crystals obtained were significantly imperfect, lacking the defined characteristics we aimed to achieve, in addition to the low yield of this system. Contrary to these results, the IPA:DDW system with an initial progesterone concentration of 12.5 [mg mL^−1^] presented promising outcomes. Crystals obtained through this method exhibited improved homogeneity, with a vast majority of hollow crystals. Additionally, the crystals obtained from the AcN:DDW system with an initial progesterone concentration of 16.67 [mg mL^−1^] displayed homogeneous dense characteristics, with a significantly high yield (see AcN:DDW crystallization system section). Accordingly, we decided to proceed with these two systems since both exhibited relatively homogeneous crystal habits, where the IPA:DDW system exhibited hollow crystals and the AcN:DDW system exhibited condensed crystals, in addition to the high yield, particularly for AcN:DDW system. As such, given the controlled features, the two systems, IPA:DDW and AcN:DDW, were found to be promising for developing and engineering progesterone microcrystals with the potential to be utilized for localized delivery and thus were further deeply investigated.

### 2.1 IPA:DDW Crystallization System

**Figure 3:**
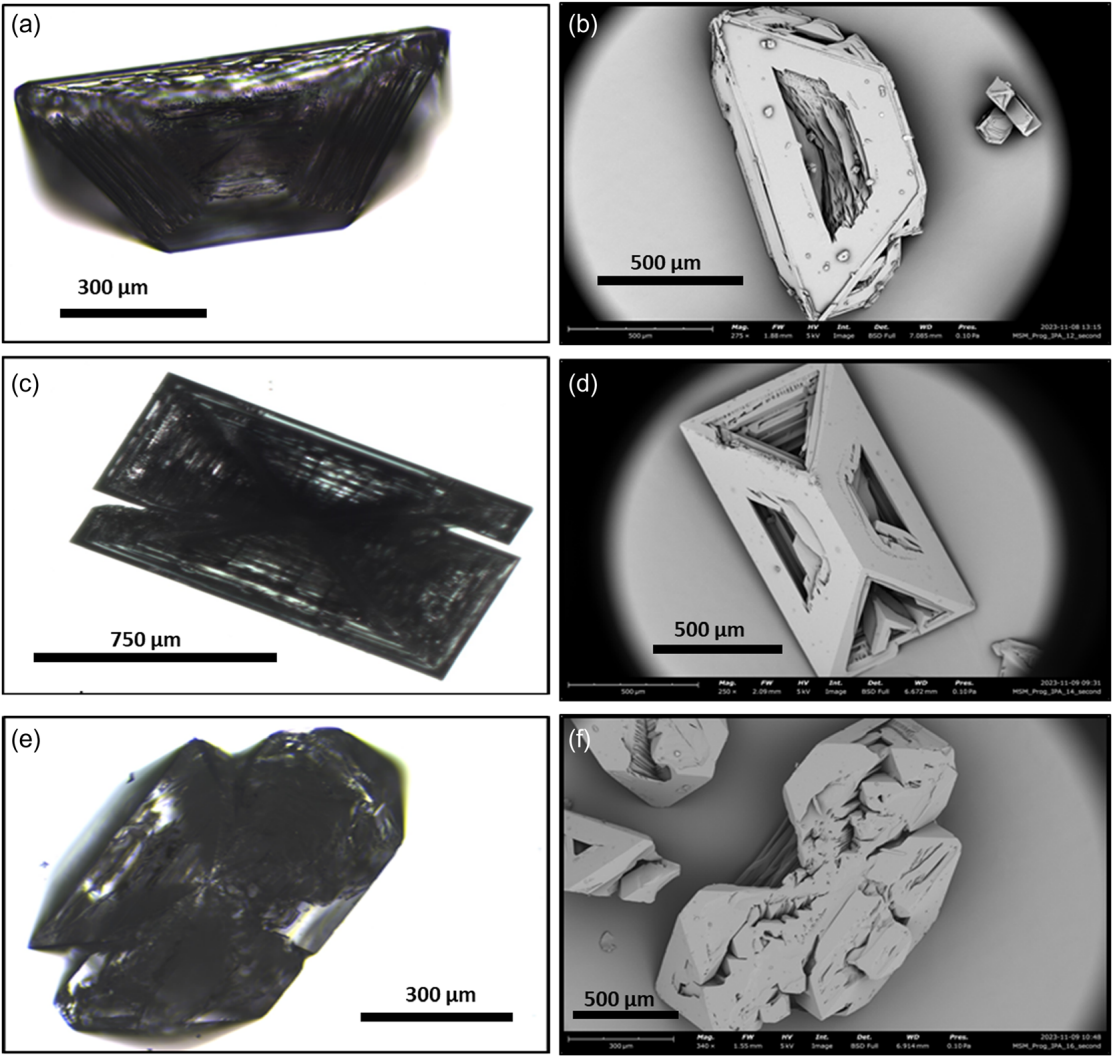
Brightfield microscopy (EVOS) (left) and SEM (right) images of the progesterone crystals prepared with IPA:DDW 1:2 solvent:anti‐solvent crystallization system with different initial progesterone concentrations: a,b) progesterone initial concentration is 12.5 [mg mL^−1^], SEM parameters include work distance (WD) = 7.065 mm and a magnification of ×275, c,d) progesterone initial concentration is 14.29 [mg mL^−1^], SEM parameters include WD = 6.672 mm and a magnification of ×250, and e,f) progesterone initial concentration is 16.67 [mg mL^−1^], SEM parameters include WD = 6.914 mm and a magnification of ×340, showing different crystals’ habits. These habits are the typical habits that coform in the IPA:DDW crystallization system, regardless of the initial solution concentration. More crystal habits are presented in Figure S1, Supporting Information. The images show hollow crystals irrespective of the initial solution concentration.

Driven by the aforementioned findings, the different parameters impacting the crystallization, including progesterone initial concentrations, solvent:anti‐solvent ratios, and crystallization temperature, were subjects to a thorough study. For the progesterone initial concentrations impact, we have used IPA:DDW for the bench crystallization technique with a 1:2 solvent:anti‐solvent ratio (v/v ratio) at 3 initial progesterone concentrations of 12.5, 14.29, and 16.67 [mg mL^−1^]. Relatively, a low crystal yield was gained when we started with a 12.5 [mg mL^−1^] progesterone concentration compared with higher initial progesterone concentrations: 14.29 and 16.67 [mg mL^−1^]. The microscopic analysis by EVOS and SEM images (**Figure** **3**) shows different hollow microcrystal habits, such as triangular prism‐like, trapezoid, and wings‐like. The presented habits in Figure 3 are the common ones and are obtained in the IPA:DDW system regardless of the initial concentration of the drug. For more crystal habits, see Figure S1, Supporting Information.

**Figure 4:**
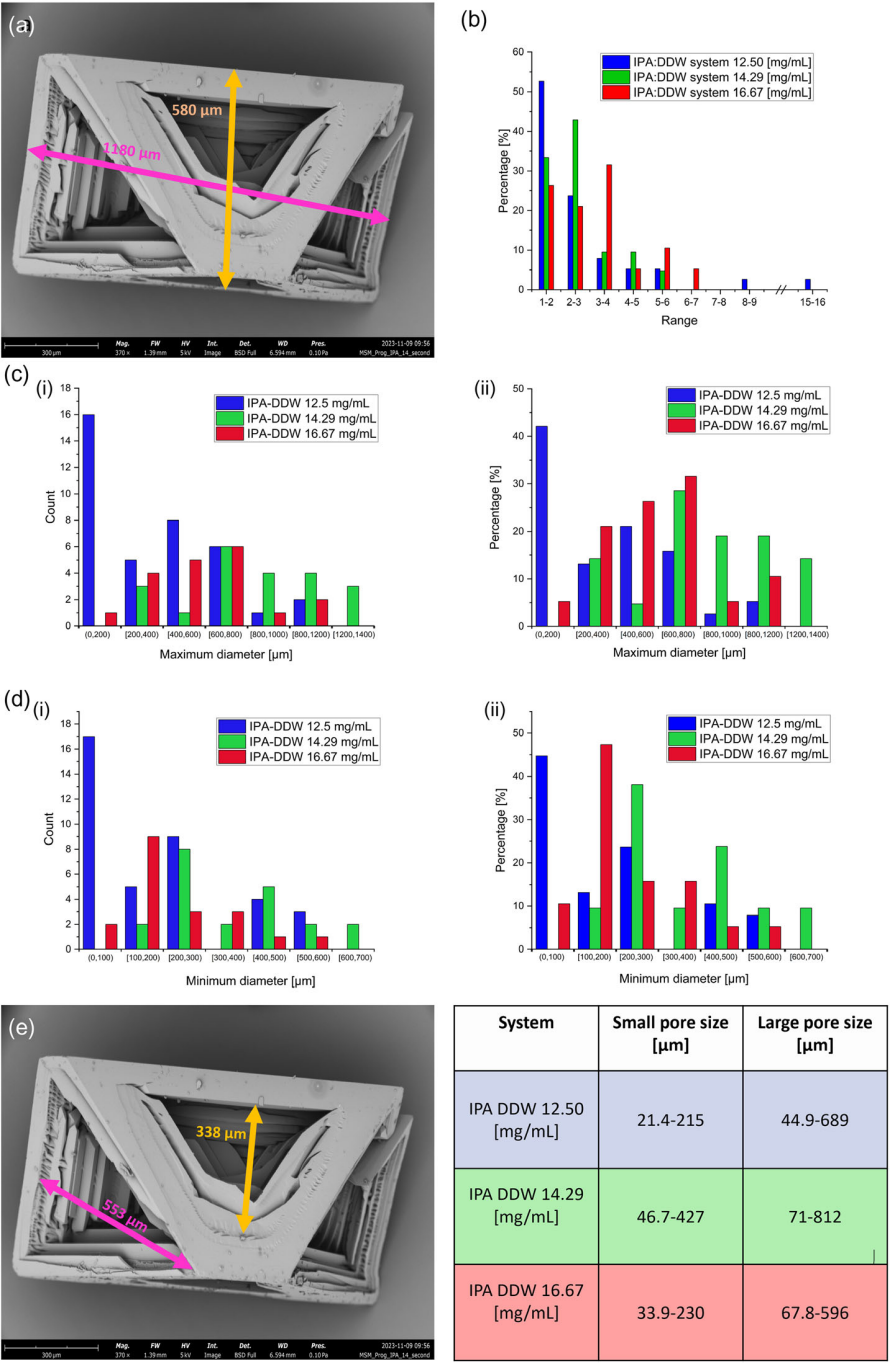
Progesterone microcrystals’ size distribution analysis: a) SEM image of the progesterone microcrystal after IPA:DDW 1:2 solvent:anti‐solvent crystallization shows the maximum (in pink) and minimum diameter measurements (in orange). SEM parameters include WD = 6.594 mm and magnification of ×370. b) The histograms depict the distribution of crystal aspect ratios calculated as the ratio between the largest and smallest crystal axis dimensions. Blue columns represent crystals from the IPA:DDW system with an initial drug concentration of 12.50 [mg mL^−1^], green columns represent crystals from the IPA:DDW system with 14.29 [mg mL^−1^], and red columns represent crystals from the IPA:DDW system with 16.67 [mg mL^−1^]. c) Histograms i and ii present the maximum microcrystal diameter by count and percentage of crystallized progesterone in a solution per initial concentration. d) Histograms i and ii present the same microcrystal's minimum diameter distribution by counts and percentage, respectively. The analysis was performed for microcrystals crystallized in a solution with initial drug concentrations of 12.5 (blue), 14.29 (green), and 16.67 (red) [mg mL^−1^]. A higher concentration of the initial drug solution yielded a narrower microcrystal size distribution. e) *Left*, the same SEM image of subpanel (a) of the progesterone microcrystal shows the large pore size (in pink) and small pore size (in orange) determination. *Right,* a summary table of pores ranges of both the small and large sizes as per crystallization system concentration.

Moreover, different crystal sizes were acquired; using SEM, we arbitrarily measured at least 19 crystals in each sample to determine their size distributions. For the best way to demonstrate the data, we first had to define two parameters, maximum and minimum diameter measurement: **Figure** **4**a. All histograms of the crystals’ aspect ratio, maximum and minimum diameter distributions, by counts and percentage, for all the preparations are shown in Figure 4b–d. According to the histograms, solutions with progesterone concentration of 12.5 [mg mL^−1^] obtained microcrystals with a wide size distribution where the central part of the crystals was relative to the other, small; while in 14.29 and 16.67 [mg mL^−1^] progesterone concentration, a narrower distribution was obtained. More importantly, all the microcrystals were hollow‐like structures, Figure 3, 4 and S1, Supporting Information. Also, a correlation was observed between the initial progesterone concentration and porosity size in the crystals where the pores size interval progressively increases with concentration, while 16.67 [mg mL^−1^] was found to decrease slightly. This might be explained due to the increased force for crystallization at higher concentrations.

**Figure 5:**
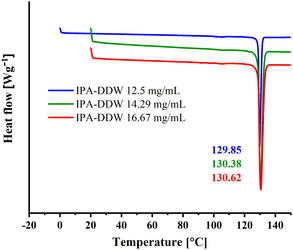
DSC analysis of progesterone crystals gained by the IPA:DDW crystallization system, using different progesterone initial concentrations: 12.5, 14.29, and 16.67 [mg mL^−1^], represented by blue, green, and red curves, respectively. The samples were weighted before analysis, with masses of 3.77 mg, 3.41 mg, and 5.07 mg, respectively. The analysis was performed with the aluminum standard 40 μl crucible. A melting peak in the temperature range of 129–131 °C from all the samples reinforces the evidence of obtaining the stable form 1 of progesterone crystals.

For further investigation of the gained microcrystals, a thermal stability test was performed by differential scanning calorimetry (DSC). The DSC results of the three types of microcrystals obtained from the different initial solution concentrations of 12.5, 14.29, and 16.67 [mg mL^−1^] are presented in **Figure** **5**, by blue, red, and green curves, respectively. The results show an endothermic peak at ≈130 °C attributed to the melting of the more stable polymorph – form 1.

To calculate the yield of each experiment, we measured the mass of the gained microcrystals and divided it by the mass of the progesterone that was added in the first place. Results are summarized in **Table** **1**. These results indicate that with a higher initial progesterone concentration, a higher yield is obtained as expected since the driving force for crystallization increases directly with the concentration.

**Table 1:** Yield of progesterone crystals in the IPA:DDW crystallization system at different initial concentrations of progesterone.

Progesterone concentration [mg mL^−1^]	Ratio of the IPA:DDW (v/v)	Average yield ± s.d. [%]
12.50	1:2	9 ± 5
14.29	1:2	16 ± 4
16.67	1:2	23 ± 5

To study the impact of temperature on the resulting crystals in IPA:DDW system at a ratio of 1:2 and initial concentration of 16.67 [mg mL^−1^], new preparations were performed with these conditions at 30 and 37 °C and compared to the preparation at room temperature. As expected, there is a significant drop in crystal yield from ≈23% to less than 2% and to 0% with increasing the temperature from room temperature to 30 and 37 °C, respectively. This can be explained by increasing the solubility of progesterone at higher temperatures in the studied system leading to less driving force for crystallization. To study the impact of solvents:anti‐solvents ratio, preparations of 1:1.5 and 1:2.5 and an initial concentration of 16.67 [mg mL^−1^] were prepared and compared to 1:2 at room temperature at the same initial concentration. It was found that in the preparation at 1:1.5, no crystals were formed due to the relatively high percentage of the solvent reducing the force for crystallization. In contrast, while the ratio 1:2.5 was anticipated to produce a higher yield and found to be above 35% due to the increase in driving force, these crystals were identified with a significant change in the habit and a decrease in porous size, Figure S2, Supporting Information.

### 2.2 AcN:DDW Crystallization System

The AcN:DDW crystallization system followed the same procedure as the IPA:DDW system. For the new crystallization system, we had to find the optimal initial drug concentration, which depends on the drug's solubility in AcN and the ratio between the solvent and anti‐solvent, which provides a sufficient driving force for crystallization. We have carried out varied initial solution concentrations and found that for drug crystallization, the initial drug concentration is 16.67 [mg mL^−1^], while the ratio between the solvent and anti‐solvent is 1:2, respectively.

**Figure 6:**
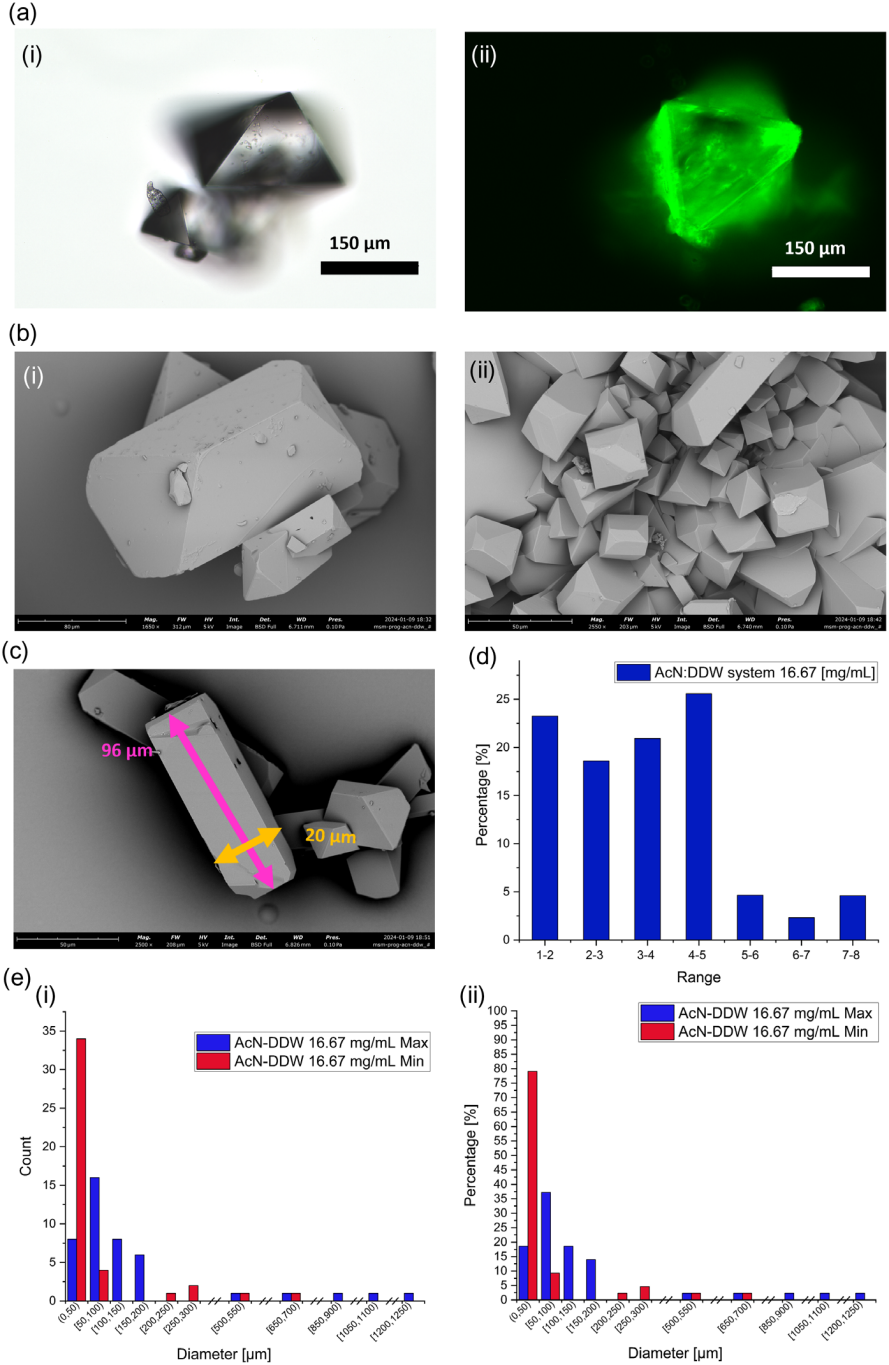
a) (i and ii) EVOS images of the progesterone crystals produced via AcN:DDW 1:2 solvent:anti‐solvent crystallization system, show different habits and sizes. b) (i and ii) SEM images of the crystals show small dense crystals (i) and crystal clusters (ii) and images were taken with WD = 6.721 and 6.740 mm, magnification of ×1450 and ×2550, respectively. c) SEM image of the progesterone microcrystal after AcN:DDW 1:2 solvent:anti‐solvent crystallization shows the maximum (in pink) and minimum diameter measurements (in orange). SEM parameters include WD = 6.826 mm and magnification of ×2500. d) The histograms depict the distribution of crystal aspect ratios calculated as the ratio between the largest and smallest crystal axis dimensions. Blue bars represent the aspect ratios of crystals from the AcN:DDW system with an initial drug concentration of 16.67 mg mL^−1^. e) Crystal's count (i) and percentage (ii) as a function of the maximum and minimum diameter show a wide distribution.

**Figure** **6** shows (a) EVOS and (b) SEM images of the microcrystals obtained with the AcN:DDW crystallization system, similar to the IPA:DDW system. Also, here, we gained different microcrystal habits, and large‐size distributions (c). Some of the microcrystals appeared clustered, and others separately, Figure 6b and S3, Supporting Information. Again, for the best way to demonstrate the data, we first had to define two parameters, maximum and minimum diameter measurement, Figure 6c. All histograms of the crystals’ aspect ratio, maximum and minimum diameter distributions, by counts and percentage, are shown in Figure 6d,e. The histogram of the microcrystals’ size distributions shows a broad distribution while the vast majority are the smaller crystals, Figure 6e. Interestingly, almost no hollow structures were observed in Figure 6a,b and S3, Supporting Information.

**Figure 7:**
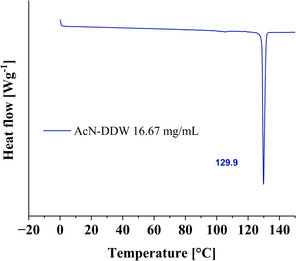
DSC analysis of progesterone crystals obtained from the AcN:DDW crystallization system. The mass of the sample used for the analysis was 3.79 mg in an aluminum standard 40 μL crucible. A melting peak is shown in the temperature range of 129–131°C, which is attributed to the melting of the stable form 1 polymorph.

**Figure 8:**
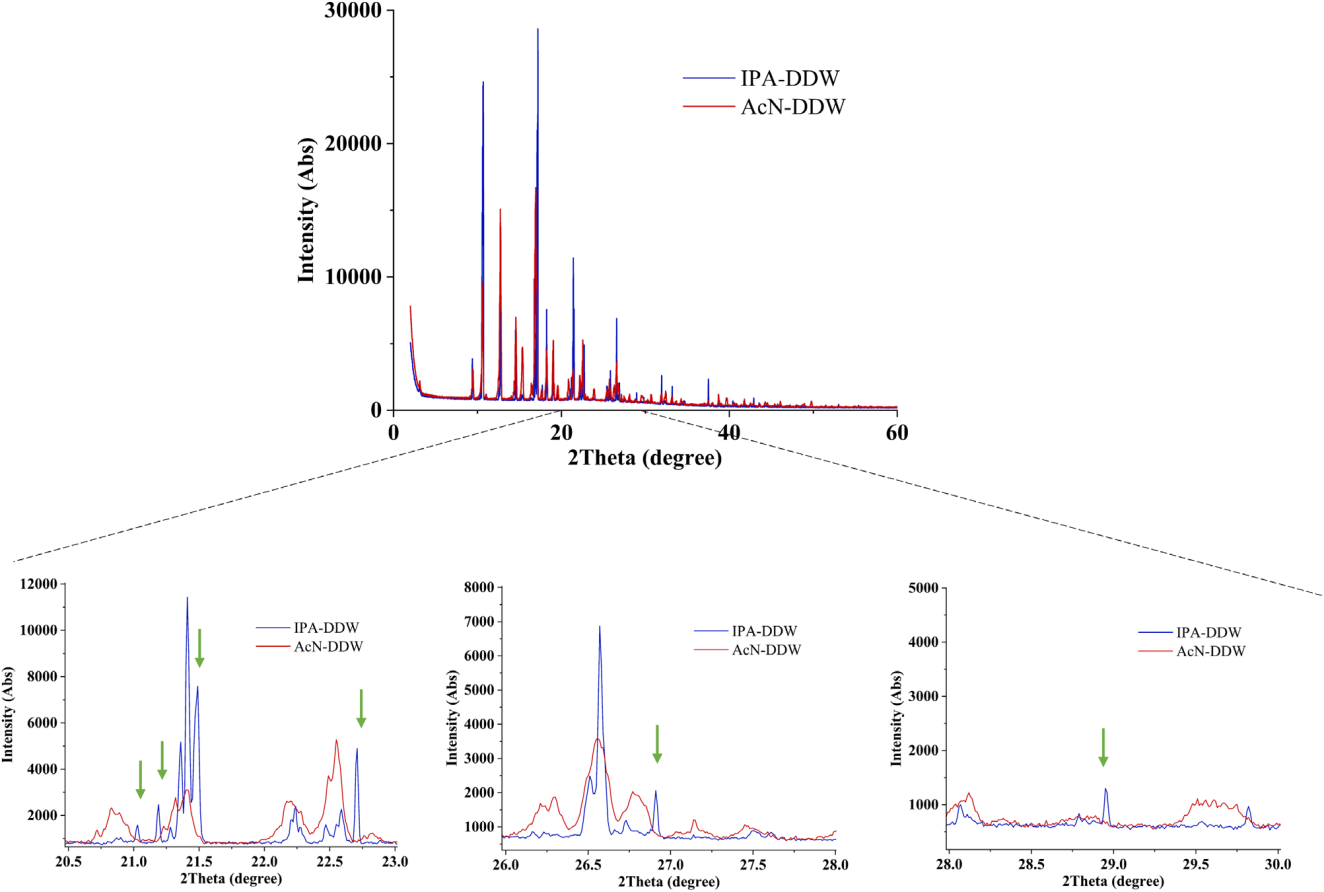
PXRD analysis of progesterone crystals, which were obtained from the AcN:DDW system presented by the red diffraction patterns, and of progesterone crystals obtained from the IPA:DDW crystallization system with an initial drug concentration of 12.5 [mg mL^−1^], given by the blue diffraction patterns. Zoom in for three angles’ regions show additional peaks for the crystals obtained from the IPA:DDW crystallization system. Note: the broader peaks of AcN:DDW can be attributed to the smaller crystal size. We can conclude from the PXRD and DSC results that the crystals obtained from both IPA:DDW and AcN:DDW crystallization systems are of the stable form 1 polymorph.

**Figure 9:**
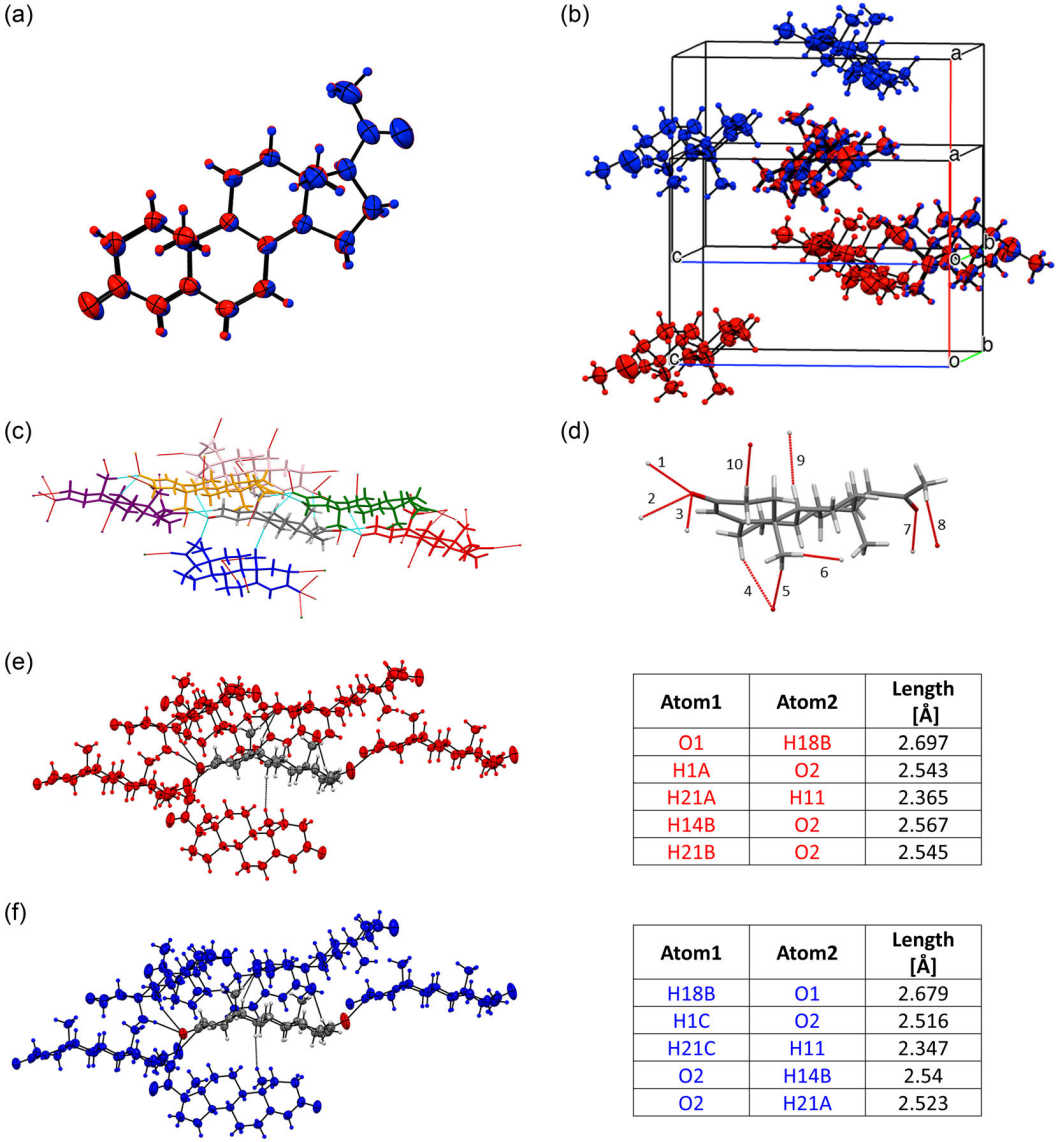
SXRD analysis of progesterone microcrystals. a) Structure comparison between AcN:DDW (red) and IPA:DDW (blue). b) The two structures have similar packing with half translation over *the a‐axis, which is a typical translation due to the symmetry of the* 2_1_ *a*‐axis. The 2_1_ symmetry element means a 180 rotation with 0.5 translation along the axis. c) The 6 surrounding molecules for each progesterone molecule. The centered molecule is colored gray, and each of the 6 surrounding molecules is colored differently: purple, red, blue, orange, green, and pink. d) The 10 interactions for each progesterone molecule. e,f) The supramolecular connectivity and, the number of surrounding molecules and their orientations match perfectly. Also, the selected interactions from both compounds exhibit excellent matching. All these findings support the conclusion that those crystals are the same polymorph.

Afterward, the microcrystal's thermal stability was tested by DSC. It showed a single sharp endothermic peak at ≈129.5 °C, which is attributed to the melting of stable form 1 polymorph, **Figure** **7**. For a profound polymorphism study, a powder X‐ray diffraction (PXRD) test was performed on microcrystals obtained from both crystallization systems, and the resulting diffraction patterns were compared as shown in **Figure** **8**. Considerable overlap between both diffraction patterns is seen, with additional peaks in the patterns of the microcrystals obtained by the IPA:DDW crystallization system. According to the progesterone database,^[^ ^22^ ^]^ both diffraction patterns are suitable to form 1 polymorph. The additional peaks in the IPA:DDW system indicate a preferred orientation. This result corresponds with the DSC result and single XRD (SXRD) analysis, **Figure** **9**, Table S3, and Figure S4, Supporting Information. See the crystal data and structure of progesterone microcrystals studied at different temperatures in Table S3 and Figure S5–S8, Supporting Information (CCDC #2345265‐2345268). In addition, the SXRD results and the unit cell parameters obtained in this study were found to be the same as the previously reported work (CCDC # 228768).^[^ ^21^ ^]^ Moreover, we have plotted predicted PXRD diffraction patterns of the developed systems versus the predicted PXRD diffraction pattern of the reported structure in the literature (CCDC # 228768) and also confirmed the same polymorph, Figure S9, Supporting Information.

Similarly to the IPA:DDW system, we measured the crystals’ yield, as shown in **Table** **2**. The yield in the AcN:DDW system was much higher than in IPA:DDW, which makes it a robust candidate system in the future in terms of progesterone crystallization yield and form specificity.

**Table 2:** Yield of progesterone microcrystals of IPA:DDW crystallization system versus AcN:DDW system at initial drug concentration 16.67 mg mL^−1^.

Crystallization system	Ratio of the solvent:anti‐solvent (v/v)	Average yield ± s.d. [%]
IPA:DDW	1:2	23 ± 5
AcN:DDW	1:2	49 ± 5

### 2.3 Release Profile

The releasing profiles of the progesterone microcrystals obtained from both studied crystallization systems were tested and compared to the releasing profile of the amorphous formula. The microcrystals from the IPA:DDW crystallization system were obtained under the crystallization conditions of an initial drug concentration of 12.5 mg mL^−1^ and a ratio of 1:2 between the solvent and the anti‐solvent, respectively. At the same time, the microcrystals from the AcN:DDW crystallization system were obtained under the crystallization conditions of an initial drug concentration of 16.67 mg mL^−1^ and a ratio of 1:2 between the solvent and the anti‐solvent, respectively. Both systems are identified by a wide distribution of microcrystals’ size, with a vast majority of relatively small microcrystals, especially the AcN:DDW system. The experiment was performed in both cases with microcrystals smaller than ≈500 μm.

#### 2.3.1 Progesterone Encapsulation

The microcrystals were prepared and suspended uniformly in the MVG alginate solution of 2% (w/v) to form drug microcrystals concentration of 10 [mg mL^−1^] in alginate solution. Then, alginate capsules were formulated using a micro‐electro‐encapsulation system with a 1 mL min^−1^ flow rate determined by the syringe pump and a voltage of ≈7.5 kV. For crosslinking the alginate, a barium chloride solution was used. In comparison, the amorphous progesterone capsules were prepared by dissolving the drug first in ethanol and then adding it to alginate solution. These capsules were prepared and tested immediately to avoid any undesired autocrystallization.

The average drug amount in capsules was determined by picking arbitrary 6 capsules and followed their dissolution in AcN for drug extraction. High‐performance liquid chromatography (HPLC) tested the solution, and the drug amount was calculated according to a preprepared calibration curve. Based on these calculations, the number of capsules from each formula to start with was determined, and in our study it was set to initial drug mass of 0.48 ± 0.01 mg. A triplicate of each formula was tested. The releasing media of PBS with 0.2% sodium dodecyl sulfate (SDS) was used for accelerated release conditions, as previously reported by Farah et al.^[^ ^4^ ^]^ The release media volume at sink condition was determined to be 12 mL based on the solubility limit of the progesterone in this release media at 37 °C. The time points for releasing media with total replacement were 10 and 30 min, 1, 2, 3, 6, and 12 h, 1, 2, 3, 4, 5, 6, 7, 11, and 14 days.

**Figure 10:**
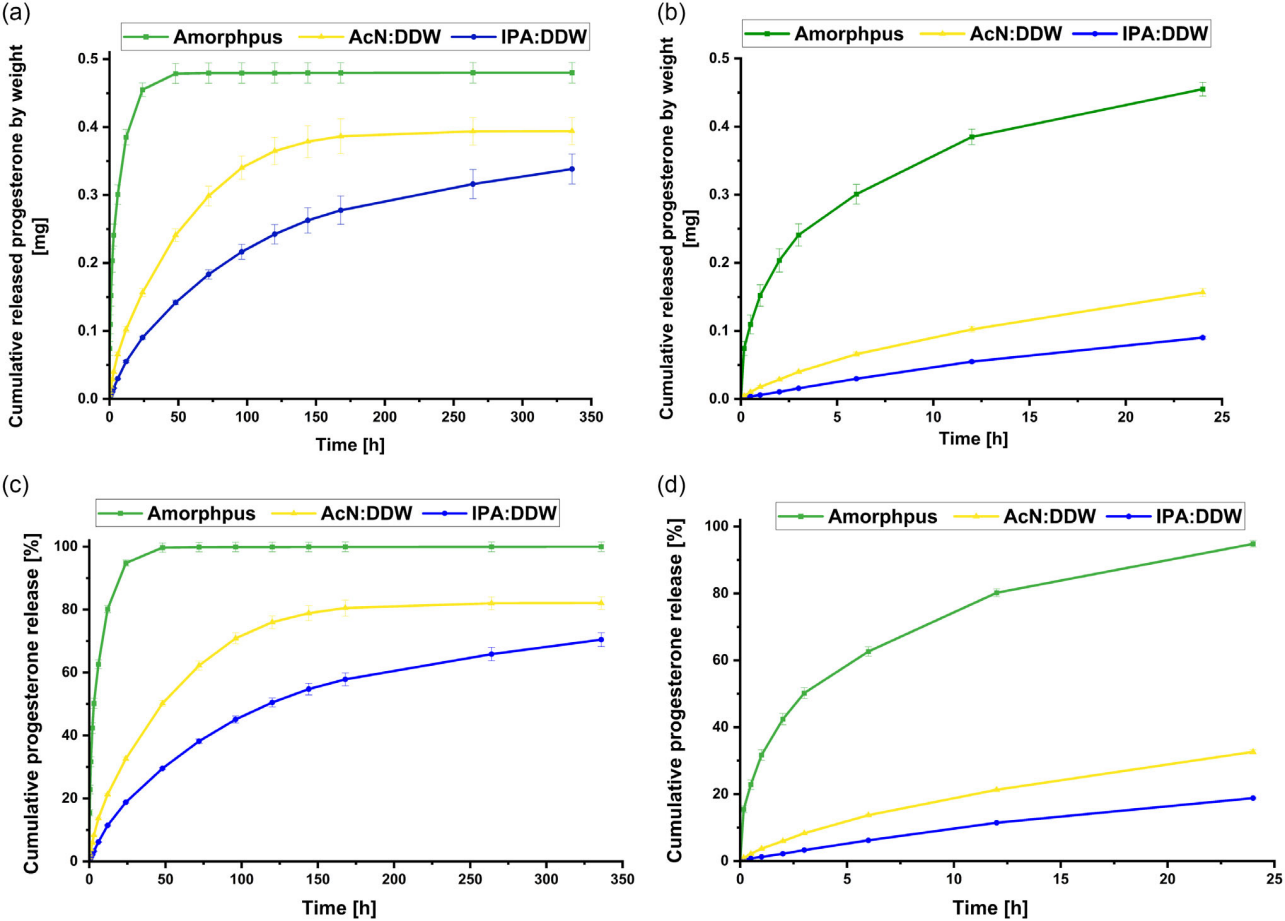
Cumulative releasing profiles of progesterone from crystals obtained from both crystallization systems versus the amorphous drug formula freshly prepared before the release study starts. a) Averaged cumulative weight release as a function of time, where the green, blue, and yellow curves represent the amorphous, IPA:DDW, and AcN:DDW formulas, respectively. b) Averaged cumulative weight release over the initial 24 h as a function of time. c) Cumulative progesterone release % as a function of time, where the green, blue, and yellow curves represent the amorphous, IPA:DDW, and AcN:DDW formulas, respectively. d) Cumulative progesterone release % over the initial 24 h as a function of time. The results show a prompt release of the amorphous formula over the first day. At the same time, the newly developed system, AcN:DDW, offers a controlled and elongated release profile of the crystalline formulas that cumulatively are 14 days higher than the IPA:DDW and lower from the amorphous form, thus offering a new level of release.

**Figure 11:**
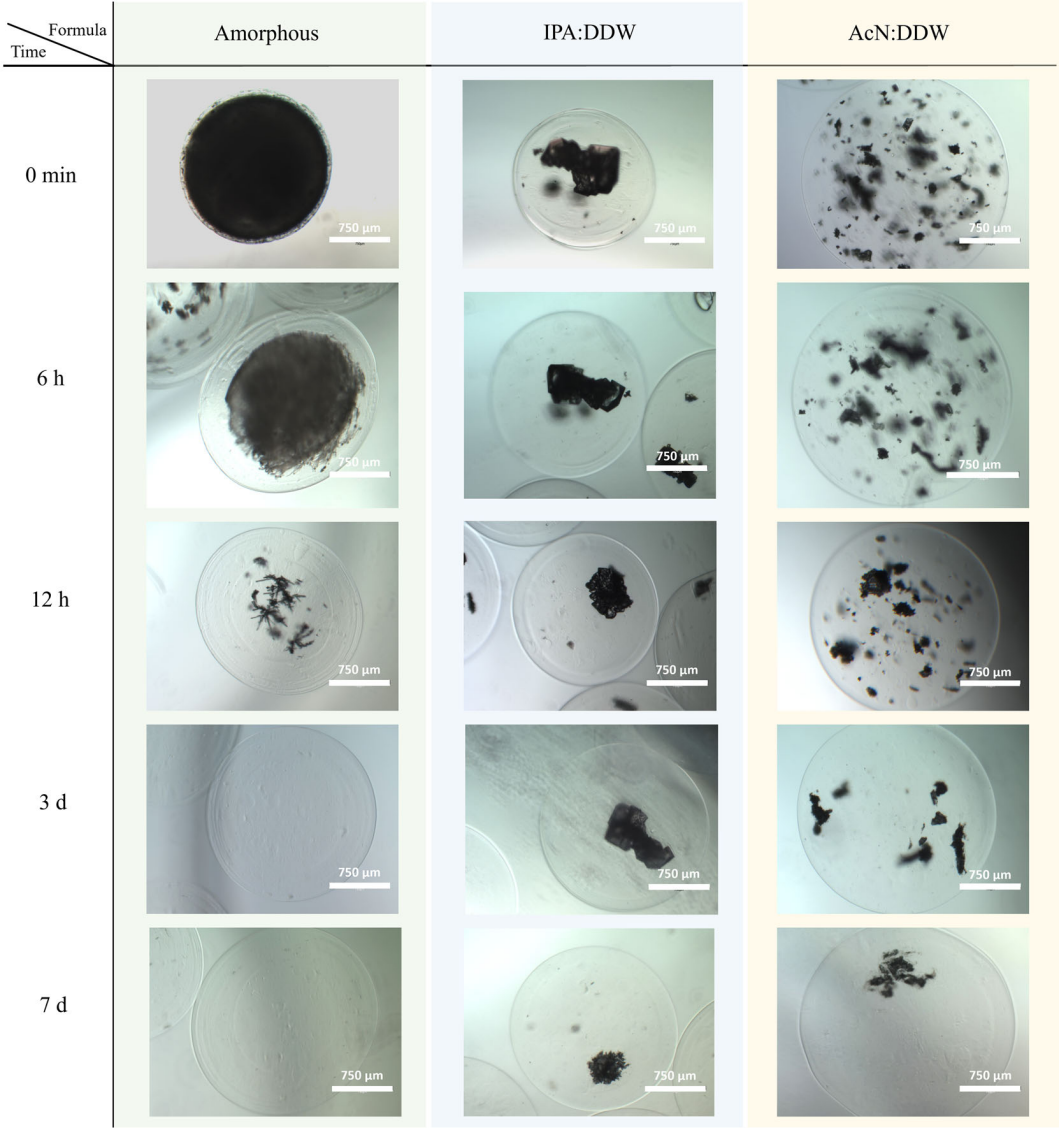
EVOS brightfield images aim to microscopically monitor the release of progesterone from alginate capsules from crystalline progesterone and its amorphous phases. The crystalline phase was obtained from the IPA:DDW and AcN:DDW crystallization systems at 0, 6, and 12 h and 3 and 7 days. The capsules were incubated at 37 °C with 50 rpm in a releasing medium of PBS with 0.2% SDS (w/v). The initial mass of the crystals encapsulated and tested in each vial was 0.48 ± 0.01 mg. The images show that the amorphous formula has been released promptly, and the capsules were found empty after 3 days. The capsules of the crystalline formulas show the crystal's partial dissolution due to the drug release from the crystals’ surface with time and maintained throughout the study.

As expected, the release results show the prompt release of the amorphous formula, where the total drug release was completed after ≈50 h, **Figure** **10**. However, the crystalline formulas showed slower and controlled release, as anticipated. This is attributed to the fact that the drug is a part of the crystalline lattice (Figure 9) and thus is released via a slow crystal surface erosion/dissolution mechanism. Nevertheless, the difference between the releasing profiles of the microcrystals obtained from the different crystallization systems can be explained by the difference in the crystal size distribution and crystal habits. This result shows the ability to choose the optimal drug‐releasing profile per individual case by using crystals obtained from the IPA:DDW crystallization system for slow release and crystals from the AcN:DDW crystallization system for a release profile that ranges between slow‐release offered by IPA:DDW and rapid‐release of amorphous profile. Moreover, combining crystals from both crystallization systems would give more options for drug release. More importantly, crystals from both systems last for 14 days, a relevant period for potential clinical application, Figure 10. These results were confirmed by microscopically monitoring the progesterone inside the alginate capsules during the study, **Figure** **11**. The images show that the amorphous formula has been released promptly, and the capsules were found empty after 3 days. The capsules of the crystalline formulas show the crystal's partial dissolution and drug release with time and maintained throughout the study, emphasizing the surface release, Figure S10, Supporting Information.

## 3 Conclusion and Future Directions

Both investigated progesterone crystallization systems yielded multiple shapes of microcrystals. The microcrystals obtained from the IPA:DDW system are hollow, whereas the ones obtained from the AcN:DDW are denser. Crystals from both systems are of form 1. However, the newly developed AcN:DDW crystallization system is more profitable than the IPA:DDW system in terms of higher yield under the same circumstances. Both systems offer a controlled release suitable to the required release period of 14 days under the expedited release model. These findings strongly indicate that these systems can offer long‐term releasing formulations that can ease drug localized administration, e.g., transvaginally, thus improving patient compliance and reducing administration issues and the needed number of doses. The success of the AcN:DDW crystallization system with progesterone gives hope that such a system can also be suitable for additional steroid hormones (e.g., estradiol) due to their chemical resemblance, and that will make it possible to combine different active pharmaceutical ingredients within one crystallization system. Such systems can offer multifunctional drug formulations that ease drug administration and solve the patient compliance administration issue. The engineered hollow crystals obtained by the IPA:iDDW system might be further used as a carrier for another steroid hormone and thus may enable sequential drug release. With that said, further hormone crystallization studies, especially the physicochemical properties of such a combination, are highly encouraged.

## 4 Experimental Section

4.1

4.1.1

##### Materials and Methods

Progesterone 98% was purchased from Acros Organics company, IPA was purchased from Bio‐Lab company, and AcN was purchased from J.T. Baker company. The PRONOVA UP MVG sodium alginate was purchased from NovaMatrix, Norway. All other chemicals and solvents were Analytical grade and purchased from Sigma‐Aldrich.

##### Crystallization of Progesterone

Progesterone solutions were prepared by weighing 30.00 ± 0.04 mg of progesterone into a vial followed by adding IPA to achieve the required initial concentration of 12.5, 14.29, and 16.67 [mg mL^−1^]; accordignly we added 2.4, 2.1, and 1.8 mL IPA, respectively, while added 1.8 mL AcN to achieve the 16.67 [mg mL^−1^] concentration. The solution was mixed using a vortex until it was clear and homogenous. Afterward, a sufficient amount of DDW was added as the anti‐solvent using a micropipette to achieve the 1:2 solvent:anti‐solvent ratio (v/v). The vials were covered with aluminum foil and constantly left at room temperature (lab‐controlled temp, 23 ± 1 °C) for 5 h. The next step was to aspirate the solvent:anti‐solvent mixture, followed by numerous crystal washing cycles with DDW. The crystals were left in the vial in the chemical hood overnight to fully dry.

##### Crystals Characterizations: Habit and Size: EVOS Microscope

EVOS microscope is a brightfield microscopy system that eliminates the complexities of high‐end microscopy while maintaining the highest performance levels. The system utilizes LED light sources (with advanced color options) to get high‐intensity output over a short light path for the most efficient fluorophore excitation. EVOS poses an excellent method to study crystal habit. At least three images were collected with 10 random measurements for each preparation or studied parameter. Crystals were visualized by EVOS M5000 Invitrogen by Thermo Fisher Scientific.

##### Crystals Characterizations: Habit and Size: SEM

Thermo Scientific XL G2 Phenom Desktop SEM was used to obtain detailed crystal habit, surface features, and size distribution. The crystals were distributed on a carbon tape and coated uniformly with a 7 nm thick gold layer to prevent charging. The electron beam intensity (5 kV), work distance (6.594–7.229 mm), magnification (×205–×3300), contrast, and brightness were fitted to obtain high‐resolution images. We used mainly the backscattered electron detector.

##### Polymorphs Detection: PXRD

Crystals polymorphism was first studied by PXRD. The diffraction pattern was measured by Rigaku LabVIEW 2 9 kW instrument, with a photon beam current of 200 mA and voltage of 45 kV. The PXRD experiments were carried out in a step size of 0.01° for 2Θ = 4°–60°.

##### Polymorphs Detection: SXRD

The progesterone single‐crystal was immersed in Paratone–N oil and mounted on a Rigaku Oxford Diffraction – XtaLAB Synergy‐S at 100 or 293 K. Data collection was performed using monochromatic Cu Kα radiation, *λ* = 1.54184 Å, using *φ* and *ω* scans to cover the Ewald sphere. Accurate cell parameters were obtained with the amount of indicated reflections. Using Olex2,^[^ ^23^ ^]^ the structure was solved with the olex2.solve^[^ ^24^ ^]^ structure solution program using Charge Flipping and refined with the ShelXL^[^ ^25^ ^]^ refinement package using least squares minimization. All nonhydrogen atoms were refined with anisotropic displacement parameters. The hydrogen atoms were refined isotropically on calculated positions using a riding model. Their *U*_iso_ values are constrained to 1.5 times the *U*_eq_ of their pivot atoms for terminal sp^3^ carbon atoms and 1.2 times for all other carbon atoms. Software used for molecular graphics: Mercury 4.3.1.^[^ ^26^ ^]^

##### Thermal Analysis: DSC

The crystal's thermal stability and phase transition detection were tested by DSC + 3 by Mettler Toledo by applying a heating profile from 0 to 150 °C at a heating rate of 10 °C min^−1^. The samples were weighted before analysis, with masses of 3–5 mg. The analysis was performed with the aluminum standard 40 μL crucible.

##### Progesterone Concentration and Release Measurement

The releasing profiles of the progesterone microcrystals were obtained following encapsulation in alginate and monitoring the release as detailed below where it was also compared to the releasing profile of the amorphous formula.

1) Progesterone encapsulation: An MVG alginate solution of 2% (w/v) was prepared where the progesterone microcrystals were suspended uniformly to form a final concentration of 10 [mg mL^−1^] in alginate solution. Then, this suspension was utilized to form 1.5 mm alginate capsules using a micro‐electro‐encapsulation system at a voltage of ≈7.5 kV with a 1 mL min^−1^ flow rate with 2.4% barium chloride solution was used as a crosslining bath. Amorphous progesterone capsules were prepared by dissolving the drug first in ethanol, followed by adding it to alginate solution, and were formulated and tested immediately to avoid any undesired autocrystallization. 2) Releasing media: PBS with 0.2% SDS at 37 °C was used for accelerated release conditions, as previously reported by Farah et al.^[^ ^4^ ^]^ Based on the solubility limit of the progesterone found by HPLC, the release media volume at sink condition was determined to be 12 mL. At each time point of the studied points, 10 and 30 min, 1, 2, 3, 6, and 12 h, 1, 2, 3, 4, 5, 6, 7, 11, and 14 days, the releasing media was totally replaced. The starting number of capsules from each formula was determined to guarantee the same initial drug amount of 0.48 ± 0.01 mg in each vail. A triplicate of each formula was tested.

##### Progesterone Concentration and Release Measurement: HPLC Parameters

HPLC is an analytical technique used to separate and quantify chemical compounds in a liquid sample. HPLC was used to test the release solutions, and the drug amount was calculated according to a preprepared calibration curve. The used HPLC instrument is by Waters, modele2695, has been used through the drug release experiment to separate, identify, and quantify the drug concentration at each time point. For this purpose, we have employed a high‐strength silica (HSS) column (XSelect HSS T3 3.5 μm), with the mobile phase being AcN/Water. The drug peak has been identified using the 2998 Photodiode Array Det by Waters at Wavelength of 243 nm and based on the retention time, where the drug concentration was quantified based on the relationship between the area under the peak and the drug concentration according to a calibration curve.

##### Statistical Analysis

All the data presented in this article are plotted and analyzed by Origin 9. Crystallization systems were repeated at least 3 times, and the yield% represents the average of 3 preparations ± SD; the crystal analysis sample size was at least *n* = 19; the release sample size was *n* = 3, and the data were presented as average ± SD.

## Conflict of Interest

The authors declare no conflict of interest.

## Supporting information

Supplementary Material

## Data Availability

The data supporting this study's findings are available from the corresponding author upon reasonable request.
