# Supplementary material for: Engineering Long‐Releasing Hollow‐like or Condensed Progesterone Hormone Microcrystals with Controlled Polymorphism
Source: Small Sci. 2024 May 26;4(8):2400045. doi: 10.1002/smsc.202400045 (PMC11935222; doi:10.1002/smsc.202400045)
Supplement: Supplementary file 1 — Supplementary Material [file SMSC-4-2400045-s001.pdf]

Supplementary Information

Engineering Long-Releasing Hollow-Like or Condensed Progesterone Hormone Microcrystals with Controlled Polymorphism

Merna Shaheen-Mualim[a†], Edwar Odeh[a†], Neta Kutner[a], Muhammad Hijazi[a], and Shady Farah[a,b]*

[a] The Laboratory for Advanced Functional/Medicinal Polymers & Smart Drug Delivery, Technologies, The Wolfson Faculty of Chemical Engineering, Technion-Israel Institute of Technology, Haifa, 3200003, Israel

[b] The Russell Berrie Nanotechnology Institute, Technion-Israel Institute of Technology, Haifa, 3200003, Israel

*Corresponding Author: Neubauer Asst. Professor Shady Farah

E-mail: sfarah@technion.ac.il

ORCID ID: 0000-0002-9801-5301

Lab website: https://www.thefarahlab.com/

Table S1 – Solubility limit of progesterone in varied solvents

Solvent	Concentration of progesterone at the solubility limit [mg/mL]	Temperature [ºC]
AcN	93	25
IPA	38	25
DMSO	47	25
Acetone	110	25
EtOH	45	25

The solubility limit of progesterone in different solvents (DMSO, EtOH, acetone, IPA, AcN) was determined using HPLC. This was achieved by adding the drug to each solvent until the solvent could no longer dissolve the drug, resulting in turbidity. Stirring was performed at 600 rpm for 3 hours using a stirrer. Subsequently, the solution was filtered, and the clear solution was diluted and tested by HPLC to determine the concentration of the progesterone at the solubility limit in the different solvents.

Table S2- Yield of progesterone microcrystals at different crystallization systems.

Crystallization system	Ratio of the Solvent:DDW (v/v)	Average yield ± s.d. [%]
DMSO:DDW	3:1	25±5
EtOH:DDW	1:2	27±4
Acetone:DDW	1:1	22±3

S1 - Crystals habit

Progesterone microcrystals obtained from both IPA-DDW and AcN-DDW crystallization systems exhibited multiple crystal habits, as shown in Figures S1 and S2. An example of a crystal's maximum and minimum diameter measurement is presented in Figure S3.

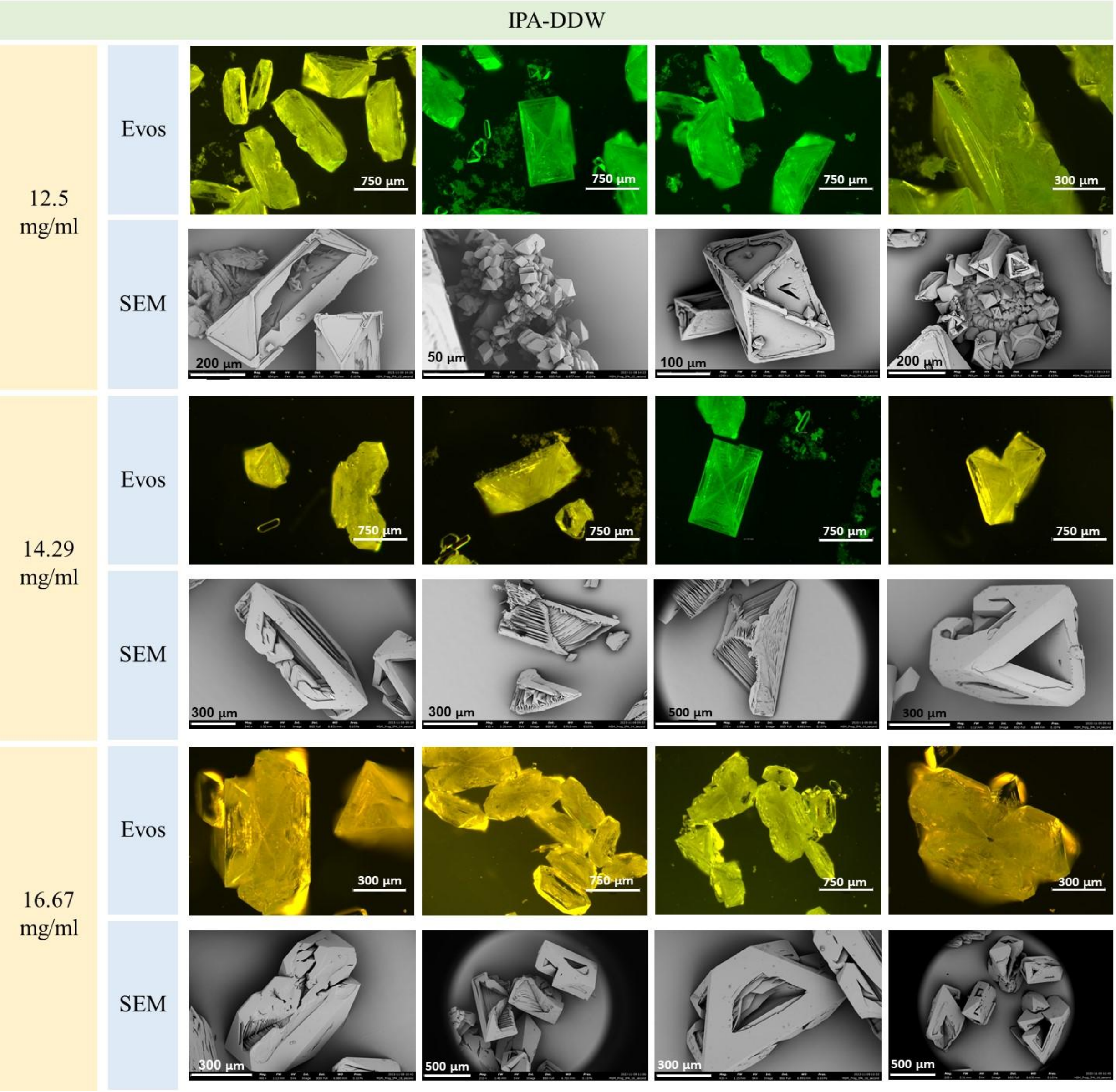

Figure S1 - EVOS and SEM images of the progesterone microcrystals after IPA-DDW 1:2 solvent-antisolvent crystallization in different progesterone concentrations: 12.5 [mg/mL], 14.29 [mg/mL], and 16.67 [mg/mL], SEM parameters include a working distance (WD) ranging from 6.630mm to 6.991mm and magnifications from x205 to x2750, showing different crystals' habits. These habits are the common habits that co-form in the IPA-DDW crystallization system, regardless of the initial solution concentration.

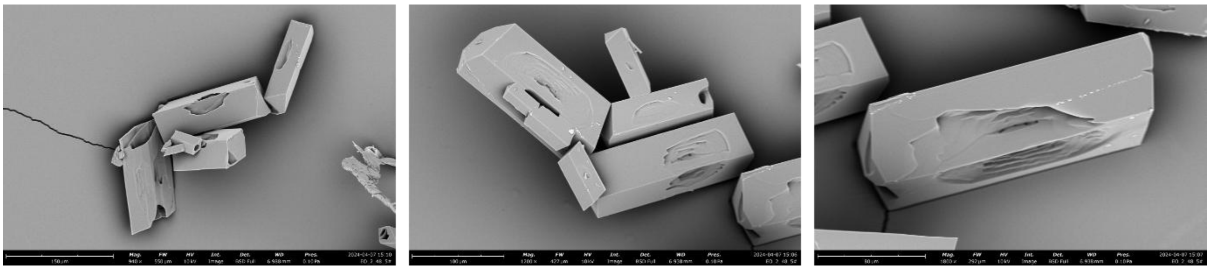

Figure S2 - EVOS and SEM images of the progesterone microcrystals after IPA-DDW 1:2.5 solvent-antisolvent crystallization at initial progesterone concentration of 16.67 [mg/mL], SEM parameters include a working distance (WD) ranging from 6.988 mm to 6.938 (both, *middle* and *right*) mm and magnifications from x940, x1200 and x1800, showing different crystals' rode-like hollow habits with decrease in pores size and porous orintations.

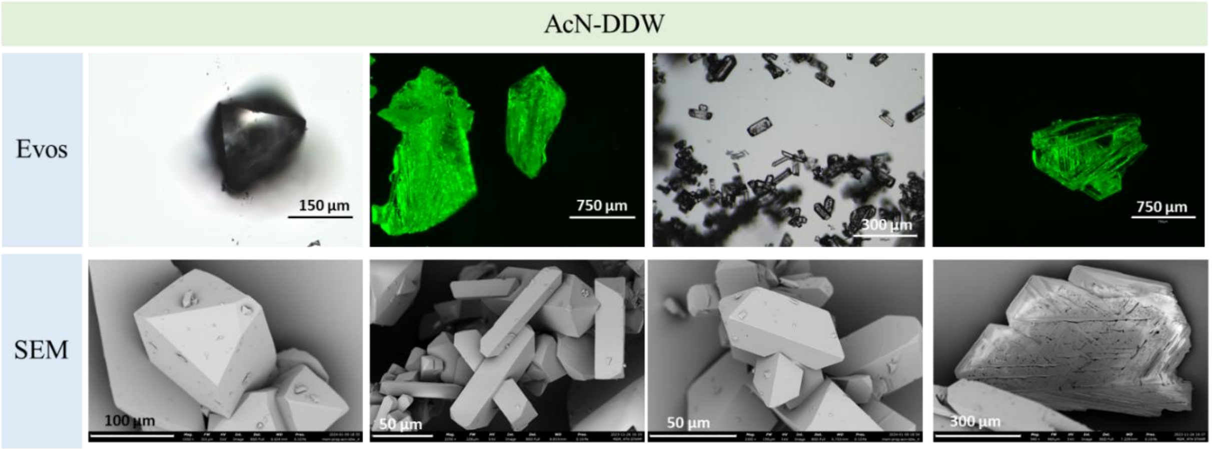

Figure S3 – EVOS and SEM images of the progesterone microcrystals after AcN-DDW 1:2 solvent-antisolvent crystallization show different habits and sizes.

S2 - SXRD

SXRD measurement was performed on crystals with different habits from IPA-DDW and AcN-DDW crystallization systems. All the measurements revealed the same parameters known for form 1 (table S1). The molecular structure of progesterone inside the unit cell and the molecular structure of progesterone are presented in Figures S5a and S5b, respectively. See the crystal structure of progesterone microcrystals studied at different temperatures Figures S5-S8.

Table S3 – Crystal data from SXRD showed the same crystal properties for both crystals obtained by the two crystallization systems

Crystal structure determination of progesterone microcrystals

Crystal data	**Shady12R from MeCN:DDW**	**Shady13R from MeCN:DDW**	**Shady14R from IPA:DDW**	**Shady11R from IPA:DDW**
Empirical formula	$C_{21}H_{30}O_2$	$C_{21}H_{30}O_2$	$C_{21}H_{30}O_2$	$C_{21}H_{30}O_2$
CCDC numbers	2345265	2345266	2345267	2345268
Formula weight	314.45	314.45	314.45	314.45
Temperature (K)	293.15	100.15	293.15	100.15
Wavelength (Å)	0.71073	0.71073	0.71073	0.71073
Crystal system,	orthorhombic	orthorhombic	orthorhombic	orthorhombic
space group	$P2_12_12_1$	$P2_12_12_1$	$P2_12_12_1$	$P2_12_12_1$
a (Å)	10.34(8)	10.23(5)	10.26(2)	10.23(4)
b (Å)	12.55(2)	12.47(6)	12.50(3)	12.47(5)
c (Å)	13.79(9)	13.60(6)	13.73(3)	13.61(5)
alpha	90	90	90	90
beta	90	90	90	90
gamma	90	90	90	90
Volume ($Å^3$)	1789.3(2)	1735.77(14)	1761.1(6)	1737.45(11)
Z	4	4	4	4
Calculated density (gr/cm^3)	1.167	1.203	1.186	1.202
Absorption coefficient (mm^{-1})	0.073	0.075	0.074	0.075
F(000)	688.0	688.0	688.0	688.0
Crystal size (mm)	0.21 × 0.15 × 0.12	0.21 × 0.15 × 0.12	0.21 × 0.18 × 0.12	0.18 × 0.15 × 0.15
2Theta range	4.924 – 59.884	4.982 – 59.692	4.956 – 59.996	4.98 – 59.732
Reflection collected/unique	10768/3951	10272/3878	11096/4008	11177/3851
Rint	0.0374	0.0519	0.0561	0.0299
Completeness (%)	99.8	100	99.8	100
Absorption correction	semi-empirical	semi-empirical	semi-empirical	semi-empirical
Data/restraints/ parameters	3951/0/211	3878/0/211	4008/0/211	3851/0/211
Goodness-of-fit on F^2	1.013	1.018	0.967	1.065
R1, wR2	0.0440, 0.1005	0.0431, 0.0993	0.0466, 0.0466	0.0363, 0.0897

[I>2sigma(I)]				
R1, wR2 (all data)	0.0662, 0.1098	0.0551, 0.1039	0.0840, 0.1064	0.0427, 0.0928
Largest diff. peak and hole	0.11/-0.17	0.32/-0.22	0.13/-0.14	0.26/-0.23
Flack parameter	-0.06(10)	0.03(9)	0.03(10)	0.03(6)
Diffractometer	XtaLAB Synergy-S	XtaLAB Synergy-S	XtaLAB Synergy-S	XtaLAB Synergy-S

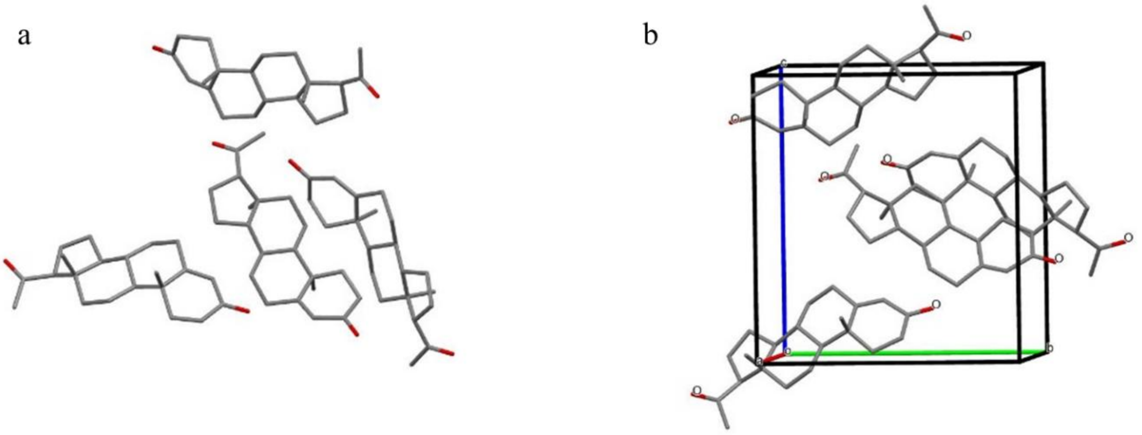

Figure S4 - The molecular structure and the molecular structure of progesterone form inside the unit cell are presented in Figures S4a and S4b, respectively.

Crystal structure of Progesterone

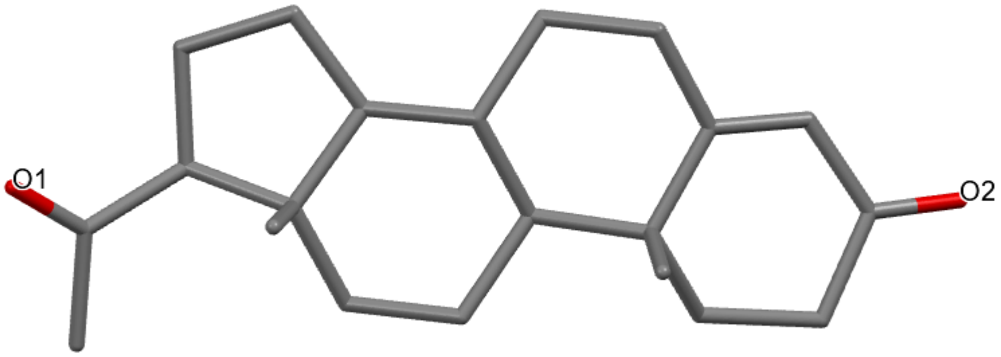

Figure S5 - IPA:DDW at r.t.

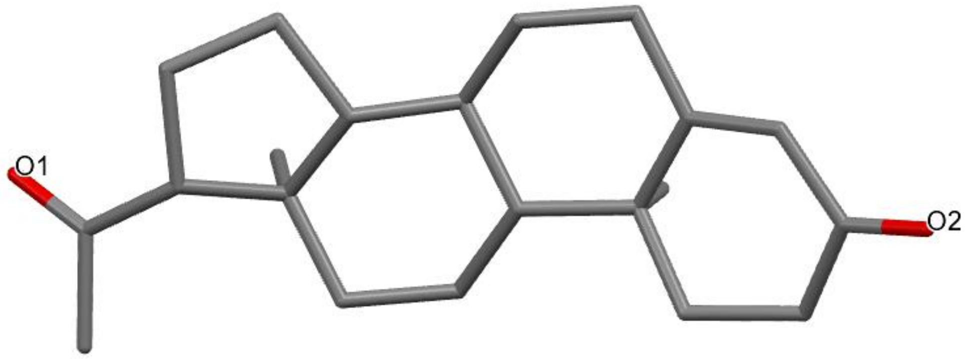

Figure S6 - IPA:DDW at 100 K

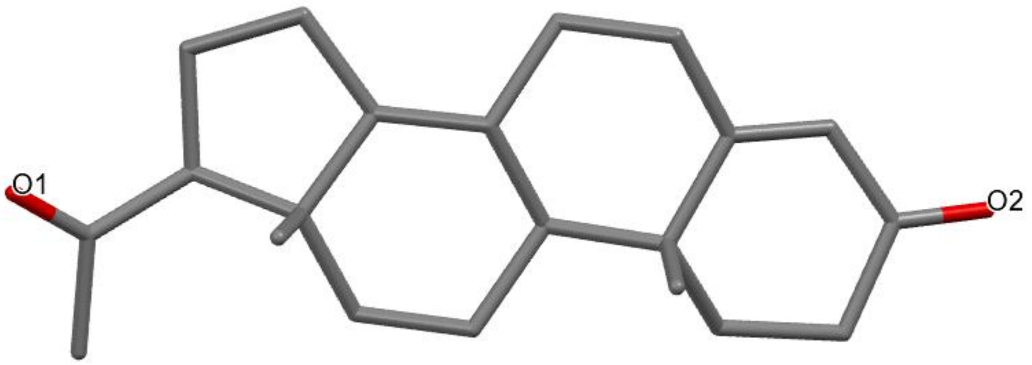

Figure S7 - MeCN:DDW at r.t.

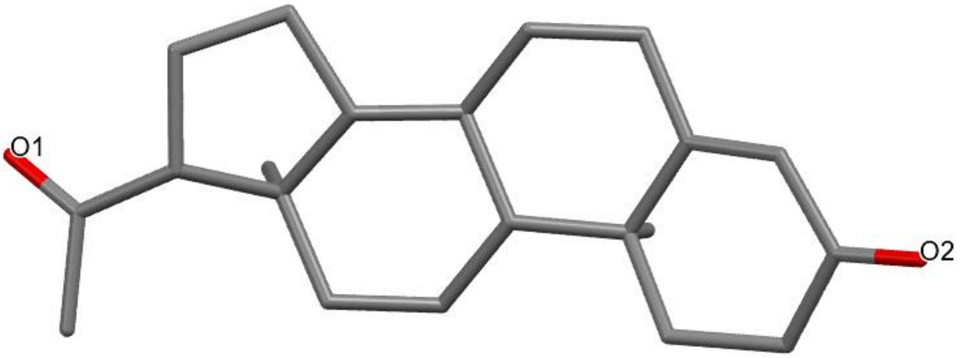

Figure S8 - MeCN:DDW at 100K

The single-crystal of colorless block materials IPA:DDW (100K) & IPA:DDW (RT) from IPA: water mixture, immersed in Paratone–N oil and mounted on a Rigaku Oxford Diffraction - XtaLAB Synergy-S at room temperature and 100K, respectively. The single-crystal of colorless block materials AcN:DDW (100K) & AcN:DDW (RT) from MeCN: water mixture, immersed in Paratone–N oil and mounted on a Rigaku Oxford Diffraction - XtaLAB Synergy-S at room temperature and 100K, respectively. Data was collected using monochromated Mo Kα radiation, $\lambda = 0.71073$, using φ and ω scans to cover the Ewald sphere. Accurate cell parameters were obtained with the amount of indicated reflections. Using Olex2[1], the structure was solved with the olex2.solve [2] structure solution program using Charge Flipping and refined with the ShelXL[3] refinement package using Least Squares minimization. All non-hydrogen atoms were refined with anisotropic displacement parameters. The hydrogen atoms were refined isotropically on calculated positions using a riding model with their U_{iso} values constrained to 1.5 times the U_{eq} of their pivot atoms for terminal sp^3 carbon atoms and 1.2 times for all other carbon atoms. Software used for molecular graphics: Mercury 2022.3.0.[4]

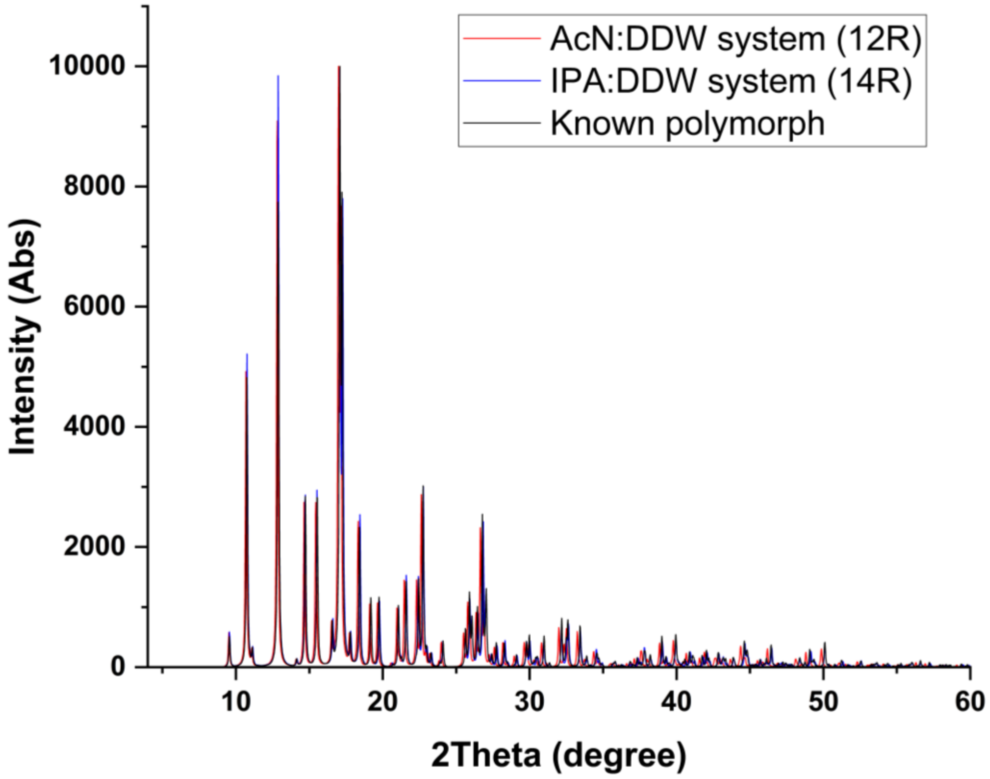

Figure S9 - Displays the predicted Powder X-ray Diffraction (PXRD) diffraction patterns derived from the Single Crystal X-ray Diffraction (SXRD) results of progesterone crystals.

The red diffraction patterns correspond to the predicted for progesterone crystals obtained from the AcN:DDW system, while the blue diffraction patterns represent predicted for progesterone crystals obtained from the IPA:DDW crystallization system with an initial drug concentration of 12.5 [mg/mL]. Additionally, the black diffraction patterns depict the diffraction patterns of the stable and known polymorph of progesterone[5].

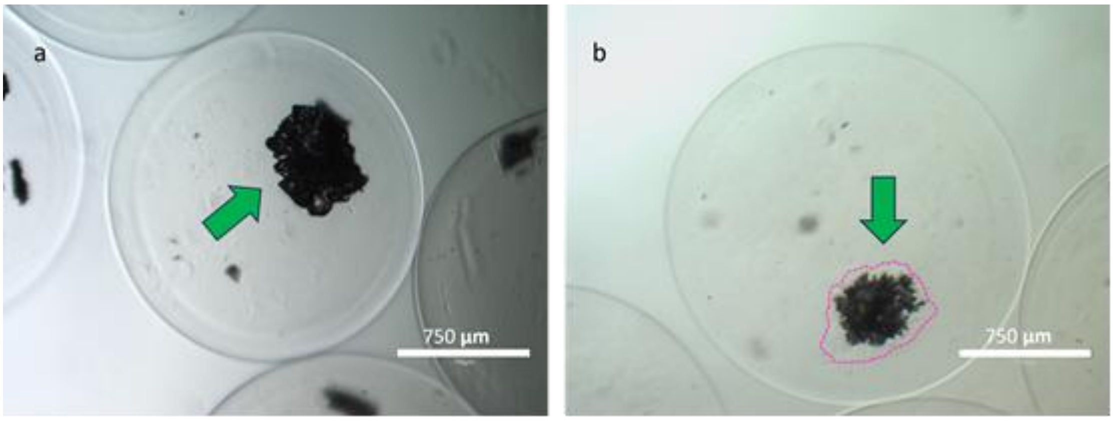

Figure S10 – Evos brightfield images aim to microscopically highlight the crystal's partial dissolution (marked with green arrows) due to surface release (marked with dashed pink line) by comparing (a) 12h to (b) 7 days under accelerated release conditions for the IPA-DDW crystallization systems. Note: these are redundant images from Figure 11 (*middle column*: 3rd and 5th rows) as examples of the partial dissolution as a function of time.

Supplementary References

1. Dolomanov, O.V., Bourhis, L.J., Gildea, R.J, Howard, J.A.K. & Puschmann, H. (2009), J. Appl. Cryst. 42, 339-341.
2. Bourhis, L.J., Dolomanov, O.V., Gildea, R.J., Howard, J.A.K., Puschmann, H. (2015). Acta Cryst. A71, 59-75.
3. Sheldrick, G.M. (2015). Acta Cryst. C71, 3-8.
4. Mercury Software from CCDC: http://www.ccdc.cam.ac.uk/ Solutions/CSDSystem/Pages/Mercury.aspx.
5. https://www.ccdc.cam.ac.uk/structures/Search?Compound=progesterone&DatabaseToSearch=Published (Deposition Number 228768, Database Identifier PROGST11)
